# Relative validation of a short questionnaire to assess the dietary habits of pregnant American Indian women

**DOI:** 10.1002/fsn3.440

**Published:** 2016-11-16

**Authors:** Terryl J. Hartman, Amy J. Elliott, Jyoti Angal, Torin Block, Erin P. Ferranti, Diane C. Mitchell, Dana C. Nickleach, Jean C. Norris, Rosalind A. Breslow, the PASS Research Network

**Affiliations:** ^1^ Department of EpidemiologyRollins School of Public Health & Winship Cancer InstituteEmory UniversityAtlantaGAUSA; ^2^ Center for Health Outcomes and PreventionSanford ResearchSioux FallsSDUSA; ^3^ NutritionQuestBerkeleyCAUSA; ^4^ Nell Hodgson Woodruff School of NursingEmory UniversityAtlantaGAUSA; ^5^ Department of Nutritional SciencesPenn State UniversityUniversity ParkPAUSA; ^6^ Winship Cancer InstituteEmory UniversityAtlantaGAUSA; ^7^ National Institute on Alcohol Abuse and AlcoholismNIHBethesdaMDUSA

**Keywords:** American Indians, diet assessment tools, dietary intake assessment, Native American, pregnancy and nutrition, prenatal nutrition

## Abstract

The objective of this study was to compare a short dietary screener developed to assess diet quality with interviewer‐administered telephone 24‐hour dietary recalls in a population of pregnant Northern Plains (NP) American Indian women. Participants were recruited from NP clinical sites of the Prenatal Alcohol and SIDS and Stillbirth (PASS) Network, as part of a large, prospective, multidisciplinary study. Prenatal PASS participants who enrolled prior to 24 weeks gestation were eligible to participate. Repeated 24‐hour dietary recalls were collected using the Nutrition Data System for Research (NDSR) software and a short dietary screener was administered intended to capture usual dietary intake during pregnancy. The available recalls were averaged across days for analysis. Items were grouped from the recalls to match the food group data estimates for the screener (e.g., total vegetables, total fruit, total dairy, total and whole grains). Deattenuated Pearson correlation coefficients were calculated between the two data sources after correcting for the within‐person variation in the 24‐hour recall data. A total of 164 eligible women completed the screener and at least two 24‐hour dietary recalls and were included in the analyses. Pearson deattenuated correlation coefficients between the diet screener and the dietary recalls for the majority of food groups were 0.40 or higher. This short diet screener to assess usual diet appears to be a valid instrument for use in evaluating diet quality among pregnant American Indian women.

## 1 Introduction

Dietary factors play important roles during pregnancy and in a wide range of maternal and fetal outcomes (Gresham et al. 2014). Suboptimal nutritional status may make the growing fetus more vulnerable to harmful environmental exposures (e.g., tobacco, alcohol, toxic chemicals) intensifying their future health consequences (Carter et al., 2012; Nykjaer et al., 2014). Alcohol has potentially deleterious effects during pregnancy (Jacobson et al., 1993). Dietary intake and quality are important considerations in studies of prenatal alcohol use because alcohol and nutrition interact at multiple levels including effects of alcohol on food intake, digestion, nutrient absorption, and metabolism (Weinberg, 1984). Studies of the effects of alcohol consumption on pregnancy outcomes and subsequent childhood growth and development should incorporate valid prospectively collected assessments of prenatal diet that address diet quality as a multi‐dimensional exposure.

There has been a shift from examining the role of single nutrients in health outcomes to assessing food patterns and overall diet quality. Diet quality varies among pregnant women by demographic and psychosocial characteristics and has been inversely associated with risk of chronic disease and more recently with adverse pregnancy outcomes (Fowles, Stang, Bryant, & Kim, 2012; George et al., 2014; Rasmussen, Maslova, Halldorsson, & Olsen, 2014; Slawson, Fitzgerald, & Morgan, 2013). In Project VIVA, among 1777 pregnant women who completed a food frequency questionnaire (FFQ), poorer quality diets were more common among those who were less educated, multiparous and who had higher prepregnancy body mass index (BMI kg/m^2^) (Rifas‐Shiman, Rich‐Edwards, Kleinman, Oken, & Gillman, 2009). These results suggest that assessment of nutritional status and overall diet quality may be particularly important among those at risk for adverse pregnancy outcomes.

There is a paucity of published nutritional data for American Indians, particularly for women of childbearing age. The majority of studies have been with at‐risk patient populations, children, or through Strong Heart, a large longitudinal study focused on cardiovascular disease (Brown et al., 2013; Eilat‐Adar et al., 2013; Fretts et al., 2012; Kattelmann, Conti, & Ren, 2009; Watts, Rockett, Baer, Leppert, & Colditz, 2007). This gap is concerning given the important role of nutrition during the prenatal period and the increased incidence of gestational diabetes and infant mortality and among American Indian populations (Acton et al., 2002; O'Connell, Yi, Wilson, Manson, & Acton, 2010). Accurate, inexpensive dietary data collection instruments that include culturally relevant foods, are acceptable to participants and which can be administered fairly quickly in a public health setting are needed for nutritional assessment. Our objective was to administer a short dietary screener to pregnant women to estimate intakes of selected food groups and to assess relative validity of the screener compared with similar measures obtained from repeated 24‐hour dietary recalls.

## 2 Methods

The Prenatal Alcohol and SIDS and Stillbirth study (PASS) is a large, prospective multidisciplinary study designed to evaluate the association between prenatal alcohol exposure and stillbirth and sudden infant death syndrome. PASS recruited women at risk for drinking during pregnancy at sites in the US Northern Plains (NP) and in Cape Town, South Africa between August, 2007 and January 2015 (Dukes et al., 2014). Oversight is provided by an independent advisory and safety monitoring board and numerous institutional review boards and tribal communities. The tribal communities represented have reviewed and approved the manuscript. Data presented here are from NP participants who participated in this ancillary diet screener validation study.

### 2.1 Participants

Between June 2011 and May 2013 203 potentially eligible women presenting for their first PASS prenatal visit at 20–24 weeks of gestation were asked to participate. The ancillary study was described and informed consent obtained. Participant characteristics available from the parent study data included age, gestational age, prepregnancy BMI, smoking, and educational status (Table 1).

**Table 1:** Participant characteristics for pregnant women (*n* = 164)

Variable	Mean (SD) or No. (%)
Age at first visit (yrs.)^a^	27.0 (5.5)
Gestation time at first visit (wks.)^a^	21.7 (1.2)
Prepregnancy BMI Category (No., %)	
Underweight/Normal (<25)	48 (29%)
Overweight (25‐29)	45 (27%)
Obese (≥30)	64 (39%)
Missing	7 (4%)
PrePregnancy BMI (kg/m^2^)^a^	29.9 (7.5)
BMI 20–24 weeks (kg/m^2^)^a^	31.28 (6.92)
Smoking (%)	
Never	24 (15%)
Past	16 (10%)
Any during pregnancy	90 (55%)
Missing	35 (21%)
Education (%)	
<H.S.	61 (37%)
H.S. graduate or GED	45 (27%)
>H.S. graduate	58 (35%)
Dietary Intake (from repeated 24‐hour recalls)	
Total energy intake (kcal)	2290 (819)
Protein (% energy)	15.0 (2.9)
Carbohydrate (% energy)	51.9 (6.6)
Total fat (% energy)	33.1 (5.5)
Cholesterol (mg)	321 (202)
Fiber (gm)	17.6 (6.7)

SD, standard deviation.

a Missing data for *n* = 7 for prepregnancy BMI, *n* = 4 for BMI at 20–24 weeks, *n* = 1 for age and gestation, *n* = 35 for smoking status due to participant nonresponse. Percentages may not add to 100 due to rounding.

### 2.2 Dietary intake measures

PASS developed a diet screener in collaboration with NutritionQuest (http://nutritionquest.com/), the source of the widely used Block FFQ by modifying a more comprehensive nutrition assessment previously used in the Strong Heart Dietary Study (Conti, 2008). The screener, a 44‐item instrument to assess usual dietary intake, queried quantity, frequency, and preparation of commonly consumed foods. Food items were defined to capture nutrient and food group values to support overall diet quality assessment. Study personnel from the community provided input on inclusion of traditional and commonly consumed foods for the study population (e.g., fry bread, menudo, wild game, Walleye fish, pumpkin). A table of these foods including energy content and macronutrients is available as an online supplement. Foods which were nutritionally similar were grouped into line items. Nutrient, food group values and portion sizes were derived from analysis of NHANES 24‐hour recall data (2003–2006) from women age 18–50 for the majority of foods. Previous studies, including the Strong Heart Study (Conti, 2008), have provided nutrient values for traditional foods and wild game. Estimates of intakes of saturated fat, sodium, and selected food group variables were generated including: whole and total fruit (including juice), dark green and orange vegetables, total vegetables (without legumes), whole grains, total grains, total milk and dairy, total meat, fish and poultry, eggs, oils, solid fats, added sugars, and energy intake from a combination of solid fats and added sugars. Alcohol consumption, collected in detail in the parent study, was not included. Participants completed the screener onsite using the previous 30 days as their recall period.

Participants provided information on convenient days, times and phone to communicate with them by telephone. The Penn State Diet Assessment Center attempted to complete a series of three interviewer‐administered telephone 24‐hour dietary recalls on randomly selected, nonconsecutive days, for each participant (2 week days and 1 weekend day) within two‐four weeks of completing the NP diet screener. The Nutrition Data System for Research (NDSR, Version 2012, Nutrition Coordinating Center, University of Minnesota, Minneapolis, MN) was used for analysis.

The screener is not intended to provide an overall estimate of total energy intake; thus, this validation focuses on food groups based on components from the United States Department of Agriculture's (USDA) Healthy Eating Index (HEI). The HEI is a tool originally developed in 1995 by the (USDA) Center for Nutrition Policy and Promotion (CNPP) to monitor and evaluate change in diet quality among US populations (Guenther, Reedy, & Krebs‐Smith, 2008; Guenther, Reedy, Krebs‐Smith, & Reeve, 2008; Guenther et al., 2013). Data from the recalls and diet screener were used to calculate selected food group components similar to those in the HEI 2005 and 2010: solid fruit; total fruit and juice; dark green and orange/yellow vegetables; total vegetables (without legumes); total dairy; meat, fish and poultry (without legumes, soy, nuts, or other protein sources); whole grains; total grains; saturated fat; and sodium (Guenther et al., 2014; Miller et al., 2011).

### 2.3 Statistical analysis

Statistical analysis was conducted using SAS Version 9.3. Descriptive statistics were reported for participant characteristics and compared to the parent study, the NP Cohort (*n* = 4805). The 24‐hour recalls were averaged across days for analysis. The mean, standard deviation, median, and interquartile range (IQR) of each nutrient and food group were reported for both the 24‐hour recall and PASS Screener. To assess the strength of the association between the two instruments, correlation coefficients were calculated using natural log transformed values of the dietary variables, and used to assess relative validity (Cade, Burley, Warm, Thompson, & Margetts, 2004). We calculated both Pearson and Spearman correlations with very similar results; Pearson correlations are reported for consistency with previously described results for pregnant women. In addition, deattenuated Pearson correlation coefficients were calculated using ANOVA to correct for the within‐person variation in the 24‐hour recall data (Willett & Lenart, 2013). Additional analysis was conducted by repeating the correlations on only women with three dietary recalls and our overall conclusions were unchanged. Bland‐Altman plots were also used to assess agreement between instruments across ranges of intakes (Bland & Altman, 1986).

## 3 Results

Of the 203 women targeted for this study, 187 were enrolled, and 16 declined (92.1%). Of the enrolled participants, at the conclusion of data collection 176 participants had responses to both the screener and 24‐hour recalls. We proposed a priori to exclude women with only one 24‐hour recall (*n* = 8) or with implausible total energy intakes (<500 kcal/day (*n* = 3) or >6000 (*n* = 1) kcal/day) leaving a final sample of 164 for analysis. The majority (96%) reported race/ethnicity as Native American with the remainder reporting as Caucasian. Mean age for validation study participants was 26.9 (±5.5 years) compared to 27.6 (±5.4 years) for the NP Cohort (*p* = .16). There were some differences between characteristics of the diet validation sample and the NP Cohort as a whole. A total of 62% of validation study participants (vs. 81% of Cohort members; *p* < .001) were high school graduates or had completed some education past high school. Smoking at some time during pregnancy was reported by 63% of the validation sample but just 38% of the NP Cohort (*p* < .0001). Lastly, 66% of validation study participants were overweight or obese (vs. 53% NP Cohort members; *p* < .001), congruent with overall the rate in this region. Mean total energy intake as estimated by the 24‐hour recalls was 2290 (±819) kcal/day with participants reporting percent energy intakes of approximately 15%, 52%, and 33% from protein, carbohydrate, and fat, respectively.

Table 2 shows intakes (means, SD and medians, IQR) for the recalls and the screener with Pearson correlations. The diet screener generally yielded estimates that were higher than the recalls for measures of fruit (nearly half a serving more/day) and vegetable (nearly one serving more/day) intake. In contrast, estimates for whole and total grains, sodium, solid fats and added sugars tended to be lower for the diet screener. Pearson correlation coefficients improved with deattenuation and ranged from a low of 0.31 for meat, fish and poultry to a high of 0.72 for milk. All deattenuated correlations were above 0.30; most were above 0.40 and considered satisfactory (Willett & Lenart, 2013). The lowest correlations observed were for meats, poultry, and fish (0.31), yellow/orange vegetables (0.38), whole (0.35) and total grains (0.36). Analyses including only women with three 24‐hour dietary recalls (*n* = 155) were not meaningfully different except for added sugars. The correlation for added sugar was improved when analyses were limited to women with three recalls (*r* = 0.64 for 3 recalls vs. *r* = 0.58 for 2 recalls).

**Table 2:** Comparison of food intake by 24‐hour recalls and PASS Screener (*n* = 164): means (SD), medians (IQR) and Pearson correlations

Variable	24‐Hour Recalls		Screener		Unadjusted correlation	Deattenuated correlation^c^
	Mean (SD)	Median (IQR)	Mean (SD)	Median (IQR)		
Fruit^a^						
Solid fruit	0.39 (0.51)	0.21 (0.52)	0.70 (0.54)	0.57 (0.71)	0.38	0.52
Total fruit & juice	0.90 (0.92)	0.71 (1.00)	1.30 (0.89)	1.09 (0.97)	0.43	0.62
Vegetables^a^						
Yellow/Orange	0.06 (0.11)	0.00 (0.06)	0.06 (0.08)	0.04 (0.07)	0.24	0.38
Dark Green	0.06 (0.13)	0.00 (0.05)	0.19 (0.25)	0.10 (0.22)	0.28	0.51
Total vegetables (without legumes)	1.41 (0.71)	1.28 (1.00)	2.27 (1.23)	1.96 (1.67)	0.27	0.44
Dairy^a^						
Milk	0.94 (0.86)	0.74 (1.02)	1.16 (0.95)	0.92 (1.32)	0.55	0.72
Total dairy	1.95 (1.25)	1.73 (1.30)	1.92 (1.13)	1.69 (1.51)	0.45	0.62
Eggs^b^	0.72 (0.79)	0.59 (1.14)	0.71 (0.58)	0.54 (0.65)	0.33	0.52
Meats^b^						
Meat, poultry, fish	4.10 (1.97)	3.71 (2.26)	3.65 (2.40)	3.17 (2.63)	0.13	0.31
Grains^b^						
Whole grains	2.10 (1.78)	1.74 (2.33)	1.62 (1.23)	1.27 (1.42)	0.25	0.35
Total grains	8.04 (3.35)	7.72 (3.62)	6.09 (3.28)	5.36 (4.68)	0.25	0.36
Fat & Sugar						
Discretionary solid fat (g)	48.77 (26.20)	43.28 (29.57)	49.51 (26.13)	44.47 (33.56)	0.35	0.47
Saturated fat (g)	30.21 (13.93)	28.03 (17.67)	29.74 (15.28)	26.75 (18.37)	0.42	0.53
Added sugar (g)	77.01 (47.89)	72.42 (55.55)	65.41 (42.78)	57.74 (61.53)	0.47	0.58
Dietary sodium (mg)	4018 (1539)	3727 (1765)	3312 (1629)	3015 (2042)	0.38	0.49

SD, standard deviation; IQR, interquartile range.

a Basic unit of measure is cup‐equivalents.

b Basic unit of measure is ounce‐equivalents or as stated.

c All correlations were statistically significant with the exception of the meat, poultry and fish group which was not significant (*p* = .19).

**Figure 1:**
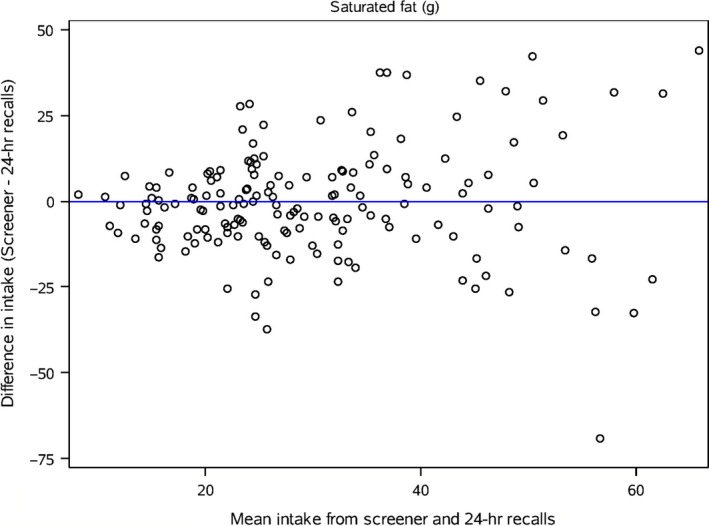
Bland‐Altman plot of the difference in intake of saturated fat between the screener and the deattenuated 24‐hour recalls plotted against the mean of the two methods

**Figure 2:**
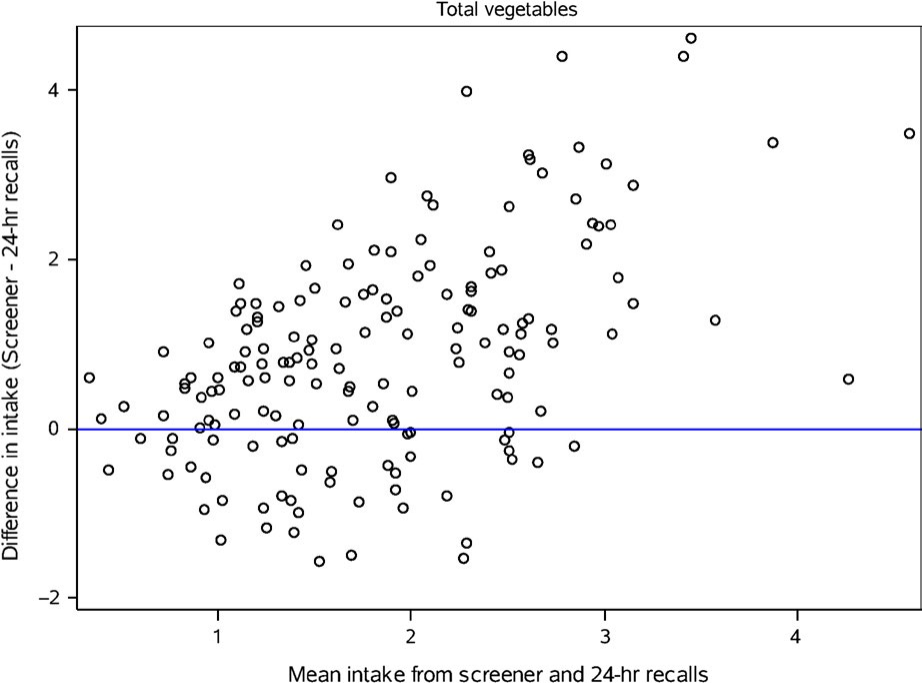
Bland‐Altman plot of the difference in intake of total vegetables between the screener and the deattenuated 24‐hour recalls plotted against the mean of the two methods

**Figure 3:**
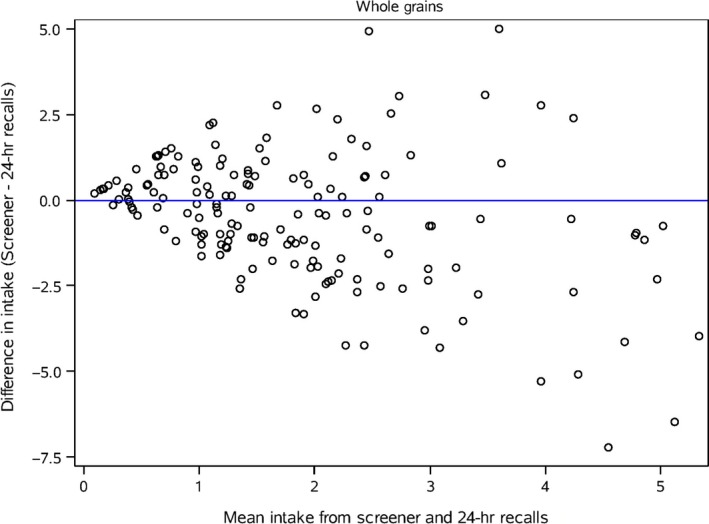
Bland‐Altman plot of the difference in intake of whole grains between the screener and the deattenuated 24‐hour recalls plotted against the mean of the two methods

Bland‐Altman plots (Figures 1, 2, and 3) of saturated fat, total vegetables and whole grains that are representative of the range in deattenuated correlation values are presented. For saturated fat agreement is fairly consistent across intakes; however, for total vegetables, the agreement is modest at higher reported intakes. The plot for whole grains does not show good agreement between methods except when reported intakes are low.

## 4 Discussion

A short diet screener was developed to assess usual intake of food groups in pregnant American Indian women. Short screener‐type instruments used to assess usual diet can be completed relatively quickly and are usually self‐administered. Compared to intakes estimated by repeated 24‐hour dietary recalls the screener provided relatively valid estimates of most food groups with correlations in the acceptable range. The lowest correlations observed were for meats, poultry and fish (0.31), yellow/orange vegetables (0.38), whole (0.35) and total grains (0.36). Generally meats are expensive; thus, it is possible that intake in this population varies on a day‐to‐day basis over the course of a month (the reference duration for the screener) and this was not captured by the dietary recalls. Another consideration for meats, poultry, and fish is that the USDA database used for the screener separates out “discretionary fat” in nonlean meats resulting in a corrected (e.g., smaller) gram amount for meats while the 24‐hour recall method does not make this adjustment. While it is difficult to determine just how this might impact the overall results, it is likely that the USDA meat measures vary from the NDSR measures in an unsystematic way. For the yellow/orange vegetable subgroup, reported consumption by both methods was very limited which likely contributed to the poor correlations; the correlation for total vegetables, which includes this subgroup, (0.44) was higher and is considered acceptable. Lastly, for grains, many of the contributors are combination foods and mixed dishes which may contribute to the difficulty in reporting usual intake frequency (e.g., for the screener) and/or portion sizes (for either method) of these foods. In addition, the USDA database used by the screener separates out fat and sugar from grains while the 24‐hour recall database does not. These differences might explain the 24‐hour recall's higher estimates for whole and total grains compared to the screener. Lastly, as explained in detail by Hunsberger, O'Malley, Block, & Norris (2015) the databases used by the two methods estimate whole grains differently. For example, if whole grain is the first ingredient in a food, NDSR considers the grain in the product whole grain. In contrast, the USDA database will assign portions of products as whole grain and nonwhole grain.

No other investigators have validated a short diet screening instrument among pregnant or nonpregnant American Indian women to our knowledge. There is one report of a validation study of an audio self‐administered computer‐assisted diet history questionnaire contrasted with monthly 24‐hour recalls among 124 American Indians and Alaska Native people (Murtaugh et al., 2010). In contrast with our study, the instrument was longer (average of 70 reported foods, required twice as long to complete), and the validation study included both genders, the majority of participants were between 35 and 49 years of age, more recall data were collected, and the focus was primarily on nutrient intake. Pearson correlations corrected for attenuation were provided for three food groups, red meat (0.67), fruits (0.32), and vegetables (0.22) (Murtaugh et al., 2010). These correlations were somewhat lower than those reported in our study for solid fruits (0.52) and total vegetables (0.44); however, our correlations for meat, poultry, fish (we did not assess red meat separately (0.31)) were lower. Our results are also comparable to those reported by other researchers for validation of diet screeners or much longer FFQs among pregnant women (Erkkola et al., 2001; Mouratidou, Ford, & Fraser, 2006; Brantsaeter, Haugen, Alexander, & Meltzer, 2008; Loy, Marhazlina, Nor, & Hamid, 2011; Barbieri, Nishimura, Crivellenti, & Sartorelli, 2013; Barbieri , Crivellenti, Nishimura, & Sartorelli, 2014). For example, Brantsaeter et al. (2008) conducted a validation study with 119 pregnant Norwegian women. Mean correlations for 4‐day weighed food diary data compared to food groups obtained from a 225‐item FFQ were 0.48 across food groups. Similar to our results, they observed correlations of 0.21 for meat and 0.48 for vegetable intakes (Brantsaeter et al., 2008).

A significant contribution of the present study was to evaluate food intake in a population of pregnant American Indian women using a short screener modified to include traditional foods. We collected prenatal diet in a prospective study design prior to examination of birth outcomes using a well‐designed protocol. The 24‐hour dietary recalls were collected by trained interviewers using state‐of‐the art methods for conducting dietary assessment research. There were also limitations to this research. Foods included in traditional American Indian/Alaskan Native diets vary by region. The screener would likely require modification to assess the traditional dietary intakes of these populations in other regions. To some extent, the diet validation substudy population differed demographically from the overall PASS population. A series of three 24‐hour recalls, often used in diet validation studies of dietary instruments, may have limited our ability to capture seasonal of intake of some traditional foods as well as “usual” pregnancy diet. In this study, both assessment methods target recent dietary intake; however, the time frame of reference between the screener and the repeated 24‐hour recalls differs somewhat which may have attenuated correlations between the two methods. The assessment of dietary intake of pregnant women could be influenced by fluctuations in appetite and by nausea, heart burn, or constipation which may be more likely to be captured by 24‐hour recalls. The diet interviewers query whether the 24‐hour recall represented a day that was typical, lower or higher than normal; however, it's possible the respondent did not feel comfortable sharing these types of diet‐related issues over the telephone.

## 5 Conclusions and Implications

This study demonstrates that the PASS Diet Screener may be a useful tool for the assessment of overall diet and diet quality among pregnant American Indian women. Future research should explore the use of this instrument with other populations.

## Conflict of Interest

TB and JN were employed by NutritionQuest; however, they did not assist with the data collection or data analysis. All other authors have nothing to report.
