# Supplementary material for: Relative validation of a short questionnaire to assess the dietary habits of pregnant American Indian women
Source: Food Sci Nutr. 2016 Nov 16;5(3):625–32. doi: 10.1002/fsn3.440 (PMC5448387; doi:10.1002/fsn3.440)
Supplement: Supplementary file 1 [file FSN3-5-625-s001.docx]

**Supplementary Table: Selected nutritional information for foods added to screener line items***

|  | **Energy (kcal)** | **Protein (g)** | **Carbohydrate (g)** | **Total fat (g)** | **Saturated fat (g)** |
| --- | --- | --- | --- | --- | --- |
| Indian tacos, 1 serving made with frybread | 560 | 33.6 | 79.7 | 12.3 | 4.8 |
| Menudo, soup, 1 C. | 118 | 12.6 | 8.0 | 3.9 | 1.2 |
| Chili stew with beef, 1 C. | 237 | 28.5 | 12.8 | 8.1 | 2.8 |
| Walleye, coated, fried in oil, 4 oz. | 229 | 23.1 | 14.2 | 8.2 | 1.31 |
| Salmon, baked/broiled with oil, 4 oz. | 213 | 28.4 | 0.1 | 10.3 | 1.7 |
| Spam, ½ in. slice | 180 | 7.6 | 2.6 | 15.1 | 5.7 |
| Venison, roasted, 4 oz. | 215 | 40.9 | 0 | 4.5 | 2.5 |
| Jerky, beef, 1 oz. | 123 | 10.0 | 3.3 | 7.7 | 3.3 |
| Bappa, buffalo meat, seasoned, oil added, 2 oz. | 120 | 22 | 1 | 1 | 1 |
| Frybread, 1 piece 7 in dia. | 391 | 8.2 | 46.0 | 19.2 | 4.9 |
| Pumpkin, ½ C. mashed | 38 | 0.9 | 1.5 | 6 | 0.3 |
| Turnips, coated, cooked in fat, ½ C. | 88 | 2.4 | 8.0 | 5.4 | 1.4 |
| Wild berries, ½ C. fresh | 31 | 1.0 | 6.9 | 0.4 | 0 |
| Wojapi, sweetened, ½ C. | 82 | 1.0 | 20.8 | 0.4 | 0 |

*Added items include both traditional foods and commonly eaten foods not included on other Block instruments.
